# Fetal alcohol spectrum disorder diagnostic clinic capacity in Canadian Provinces and territories

**DOI:** 10.1371/journal.pone.0301615

**Published:** 2024-04-03

**Authors:** Svetlana Popova, Danijela Dozet, Valerie Temple, Audrey McFarlane, Jocelynn Cook, Larry Burd

**Affiliations:** 1 Institute for Mental Health Policy Research, Centre for Addiction and Mental Health, Toronto, ON, Canada; 2 Dalla Lana School of Public Health, University of Toronto, Toronto, ON, Canada; 3 Institute of Medical Science, University of Toronto, Faculty of Medicine, Toronto, ON, Canada; 4 Factor-Inwentash Faculty of Social Work, University of Toronto, Toronto, ON, Canada; 5 Surrey Place, Toronto, ON, Canada; 6 Canada FASD Research Network, Vancouver, BC, Canada; 7 The Society of Obstetricians and Gynaecologists of Canada, Ottawa, Ontario, Canada; 8 Department of Pediatrics, North Dakota Fetal Alcohol Syndrome Center, University of North Dakota School of Medicine and Health Sciences, Pediatric Therapy Services, Altru Health System, Grand Forks, ND, United States of America; University of Montreal, CANADA

## Abstract

This study investigated the diagnostic capacity for Fetal Alcohol Spectrum Disorder (FASD) in multidisciplinary clinics across several provincial and one territorial jurisdictions of Canada: Alberta, British Columbia, Manitoba, Ontario and Northwest Territories. The data were collected directly from clinics capable of providing diagnoses of FASD and examined annual capacity for the assessment and diagnosis of FASD per year from 2015 to 2019. In total, 58 FASD diagnostic clinics were identified and 33 clinics participated in this survey. The study identified inadequate FASD diagnostic capacity in all participating jurisdictions. Based on the findings and the current population sizes, it is estimated that 98% of individuals with FASD are undiagnosed or misdiagnosed in Canada. Wait times for FASD diagnosis ranged from 1 month to 4.5 years across participating jurisdictions. The annual FASD diagnostic capacity in the select provinces and territories require at least a 67-fold increase per year.

## Introduction

Fetal Alcohol Spectrum Disorder (FASD) is a diagnostic term used to describe the impact of prenatal alcohol exposure (PAE) on an individual’s physical, cognitive, and behavioural development. The effects of PAE can vary a great deal depending on factors such as the dose and timing of exposure, genetic factors, maternal health and well-being, and other pre and perinatal exposures [1–3]. FASD is a lifelong disability and in many countries including Canada, the United States and the United Kingdom it is the leading known cause of developmental disorders [4–6]. FASD can also be considered as the encompassing term for multiple categorical entities (fetal alcohol syndrome, partial FAS, alcohol related neurodevelopmental disorder, neurodevelopmental disorder associated with prenatal alcohol exposure and multiple categories in the 4-digit code system) [7].

The underlying cause of FASD, maternal alcohol consumption during pregnancy, can be prevented in many cases, and this makes it an important target for public health initiatives. A variety of prevention efforts are possible including public awareness campaigns, pre-conception educational outreach, screening during prenatal care, access to brief interventions for pregnant women, and substance abuse treatment programs for women with addiction issues. Accurate FASD diagnosis among women of childbearing age (15–49 years) is also an important avenue for prevention because in some cases FASD occurs within a cycle where mothers who have experienced PAE themselves go on to have alcohol-exposed pregnancies [8, 9]. As well, women who have had one alcohol exposed pregnancy are at greater risk of having additional alcohol exposed pregnancies, increasing the likelihood of FASD recurrence within families [8, 10]. Identification of PAE and diagnosis of FASD may therefore be an effective method for specifically targeting families in greater need of supports and ultimately reducing the incidence of FASD. FASD diagnosis is also the starting point for developing service and support systems for those affected by PAE. Without diagnosis, there is limited understanding of prevalence and service burden, and therefore no documentation or description of service need, and no impetus to create holistic services and interventions for individuals living with FASD [11]. For this reason, considering the development of FASD diagnostic resources and supports concurrently with implementation of prevention efforts will help produce forward momentum in all areas.

To inform public policy and investment in appropriate resources for FASD diagnosis requires an understanding of the prevalence of the disorder and the existing capacity to diagnose. In terms of prevalence, a population-based study undertaken in Canada found rates of between 2% and 3% for the full spectrum of FASD diagnoses [6, 12]. A less conservative estimate of 4% is also reported in Canada [13]. Using the more conservative rate of 2.5% means there are currently about 973,247 people (any age) in Canada with FASD [14]. Of note, however, the life expectancy of individuals with FASD is suggested to be shorter than individuals in the general population, so this number may be smaller, the majority of individuals with FASD at any age are undiagnosed or misdiagnosed [15], and clinics may have limited capacity to assess and diagnose elderly populations.

The capacity to diagnose FASD has also been previously studied in Canada. Clarren and colleagues (2011) [16] found that the number of multidisciplinary clinics identifying themselves as providing FASD diagnoses in Canada in 2005 was 55 [16]; however, this number had dropped to 44 by 2011, suggesting a decrease in capacity over this period. The authors calculated capacity for FASD diagnosis per 10,000 individuals across Canada in 2011 as being .64, indicating that less than 1 diagnostic slot per 10,000 people was available in the respective year. Another recent paper also reported limited FASD clinical capacity in Western/Northern, Central and Atlantic Canada [17].

The purpose of the current study was to investigate the FASD diagnostic capacity across several provincial jurisdictions in Canada from 2015–2019. Specifically, the objectives of this research were to study: (a) the current capacity for FASD diagnosis; (b) if that capacity has increased or decreased over time; and (c) the level of service required to meet the current need, based on updated prevalence estimates of FASD. This study collected information directly from clinic coordinators of FASD diagnostic clinics regarding the number of individuals assessed for and diagnosed with FASD per year from 2015 to 2019 among multidisciplinary clinics capable of providing diagnoses of FASD in each select provinces and territories.

## Materials and methods

This was a retrospective cross-sectional study of diagnostic clinics in certain provincial jurisdictions in Canada in 2015–2019. This study was approved by the Centre for Addiction and Mental Health (CAMH) Research Ethics Board (REB #: 014/2017-04). Data were submitted by clinic coordinators, not containing any patient or clinic identifiers. A list of neurodevelopmental clinics or other clinics that might capture diagnoses of FASD in Alberta (AB), British Columbia (BC), Manitoba (MB), Ontario (ON), and Northwest Territories (NT) for the select years was created. A survey was designed by a team of clinic coordinators, researchers and physicians to obtain information regarding diagnoses and diagnostic capacity in clinics, including:

- Number of slots allotted to FASD diagnoses each year
- Number of individuals assessed for FASD each year
- Number of individuals diagnosed with FASD each year
- Number of people on the waitlist for FASD assessment each year
- Clinic definitions of waitlists

The survey was pilot-tested in August 2018 by several clinic coordinators. Data collection began in October 2018 and was ongoing until June 2020, when data analysis was conducted. Clinic coordinators were contacted and followed up with via email and phone to obtain their willingness to participate in the survey. Information about the characteristics of each clinic was also obtained from clinic coordinators. Clinics were each assigned an alphanumeric ID which was entered into a REDCap database and used to track completeness of data during the data collection process. A small honourarium was provided to clinics to thank them for their participation. Clinics from Alberta (AB), British Columbia (BC), Manitoba (MB), Ontario (ON); and Northwest Territories (NT) agreed to participate in the survey.

Descriptive statistics were generated separately for each participating jurisdiction, for FASD assessments, diagnoses, allotted diagnostic slots and waitlist sizes among clinics who contributed data for each respective year. Qualitative data from free-text responses for the descriptions of waitlists provided were amalgamated and thematically analyzed, including themes such as waitlist time, requirements for PAE confirmation and referral processes. As summary of the themes is provided for each participating province/territory.

The number of underdiagnosed cases of FASD from birth to 18 years old per year was estimated using data from Statistics Canada for annual birth rate (18) and total population (14) in the selected provinces and territories in Canada in 2017.

Ethics approval was obtained by the Centre for Addiction and Mental Health (CAMH) Research Ethics Board (014/2017).

## Results

In total, 58 FASD diagnostic clinics were identified, of which, 33 clinics participated in this survey: 14 in Alberta; two in British Columbia, one in Manitoba, one in Northwest Territories and 15 in Ontario.

### Alberta

Between 2015 and 2019, 23 clinics were identified in AB as having the capacity to conduct neurodevelopmental assessments for FASD among children and adults. Fourteen clinics out of 23 participated in the survey (response rate 60.9%), contributing at least one year of clinic data. Seven of these clinics only accepted pediatric referrals for FASD. Table 1 presents all patient populations (children and adults) among clinics that contributed data for the respective year. On average, 31–34 patients were assessed in each diagnostic clinic per year. Among them, roughly two-thirds were newly diagnosed with FASD; between 18 and 23 patients on average per year. Based on these data, we have calculated that the mean diagnostic slots per clinic was 2.6 per month; and the mean rate of new diagnoses per month was 1.8 cases, or about one new case identified every 16.2 days.

**Table 1:** Diagnostic capacity of neurodevelopmental clinics (n = 14) in Alberta that participated in the survey (2015–2019).

YEAR OF ASSESSMENT	2015	2016	2017	2018	2019
**Number of clinics contributing data**	**7**	9	9	12	10
**Individuals assessed in each clinic for FASD diagnoses**					
Mean (SD)	32 (10.0)	31 (14.3)	34 (10.6)	31 (17.2)	32 (9.7)
Range	13–45	12–49	14–45	6–71	21–47
**Individuals diagnosed with FASD this year**					
Mean (SD)	21 (7.0)	18 (10.8)	23 (8.0)	21 (14.7)	19 (6.5)
Range	8–29	7–39	11–34	2–48	11–32
**Diagnostic slots allotted to FASD diagnoses in this timeframe**					
Mean (SD)	32 (15.1)	31 (16.8)	29 (14.9)	30 (20.2)	34 (14.7)
Range	2–48	2–48	2–48	2–72	2–50
**Individuals waiting for FASD assessment**					
Mean (SD)	103 (17.5)	120 (0)	101 (26.3)	72 (54.4)	53 (39.8)
Range	85–120	120–120	65–140	0–185	1–110

Diagnostic slots allotted to FASD diagnoses in this timeframe ranged from 29 to 34 patients per year. The number of individuals on a wait list ranged from 53 to 103 per clinic. This means that annual diagnostic capacity of clinics does not meet the demand for patients waiting to be assessed. Waiting time for current patients for FASD assessment could be from one year to three years.

Each participating clinic was also asked to describe their waitlist, and responses were varied in detail provided. Among the 13 clinics who responded to this question, two were pilot clinics who did not have a waitlist defined or organized at the time of the survey. Four clinics indicated that PAE would need to be confirmed prior to the assessment, while three other clinics indicated that such confirmation is not always necessary, and that assessment would not be delayed if PAE information could not be found. One clinic indicated a three to six-month assessment wait time for children and a minimum wait time of six months for adult patients. There was no information contributed with regards to possible referral sources (e.g., school or community agencies); it was simply noted that the completion of a referral package was necessary to be on the waitlist.

Official data indicates that Alberta had 49,970 births in 2022 [18]. If the population-based prevalence of FASD is 2.5% (mean estimate from 2–3% prevalence) [6], the annual number of new cases of FASD is 1,249 (also 3.4 cases per day or 24 per week). If the less conservative FASD prevalence rate of 4% [13] is used, the number of new cases would be 1,999 each year (also 5.5 per day or 38 per week).

### British Columbia

Two clinics were identified in BC with the capacity to conduct neurodevelopmental assessments for FASD. Both clinics contributed data corresponding to years 2015–2019. Estimates have been combined for both clinics, as they represent the majority of FASD assessments and diagnoses in the province (Table 2).

**Table 2:** Diagnostic capacity of neurodevelopmental clinics (n = 2) in British Columbia that participated in the survey (2015–2019).

YEAR OF ASSESSMENT	2015	2016	2017	2018	2019	ALL YEARS COMBINED
**Total number of individuals assessed in all clinics for FASD diagnoses**	765	613	649	629	604	**3,260**
FASD Assessments/10,000 population	1.60	1.26	1.32	1.26	1.18	N/A
**Individuals diagnosed with FASD this year**	419	343	383	334	286	**1,765**
**Diagnostic slots allotted to FASD diagnoses in this timeframe**	1,160	1,180	1,195	1,205	1,302	**6,045**
Individuals waiting for FASD assessment *	30	40	60	60	70	**260**

*Incomplete data—only one clinic contributed data

A total of 3,260 individuals were assessed for FASD in BC from 2015 to 2019, or an average of 652 per year (SD = 65.4). Among them, 1,765 individuals (54.1%) were diagnosed with FASD. An average of 353 individuals (SD = 50.5) were diagnosed each year within the two participating clinics, or about 29 cases per month. FASD referrals were accepted as part of larger programs with referral streams. One clinic noted that management of waitlists may change as clinic capacity and contract deliverables fluctuate from year to year. Wait times may vary based on complex needs and referral stream.

Based on official data, British Columbia had 42,783 births in 2022 [18]. If the population-based prevalence of FASD is 2.5% (mean estimate from 2–3% prevalence) the annual number of new cases of FASD would be 1,070 (also 2.9 new cases per day, or 21 per week). If the less conservative prevalence rate is 4% is used, the number of new cases would be 1,711 annually (also 4.7 each day, or 33 per week).

### Manitoba

One clinic in MB was identified as being capable of conducting neurodevelopmental assessments for FASD in children (under 18 years). This clinic provided data for assessment years 2015–2019 (Table 3).

**Table 3:** Diagnostic capacity of the neurodevelopmental clinic (n = 1) in Manitoba that participated in the survey (2015–2019).

YEAR OF ASSESSMENT	2015	2016	2017	2018	2019	ALL YEARS COMBINED
**Individuals assessed in clinic for FASD diagnoses**	275	273	260	247	239	**1294**
FASD Assessments/ 10,000 population	2.13	2.08	1.95	1.82	1.75	N/A
**Individuals diagnosed with FASD this year**	142	147	152	126	120	**687**
**Diagnostic slots allotted to FASD diagnoses in this timeframe**	160	300	300	300	300	**1360**
**Individuals waiting for FASD assessment**	110	120	120	120	120	**590**

There were between 239 and 275 children assessed each year, with an average of 259 assessments (SD = 15.8) per year or 22 per month. A total of 1,294 children were assessed for FASD in 2015–2019, and over half of them (n = 687; 53.1%) were diagnosed with FASD. Across all years, between 50% and 58% of assessments in each year resulted in diagnoses of FASD. In each year, there were between 120 and 150 children diagnosed with FASD, with an average of 137 children diagnosed per year (SD = 13.8) or 2.6 per day. The clinic allotted an average of 272 slots (SD = 62.2) per year to FASD diagnoses, or a total of 1360 across all years. On average, there were 118 children on the waitlist (SD = 4.5) each year; based on these data, the estimated wait time for an FASD evaluation is 5 months. Children were eligible to be on the waitlist if screening criteria were met, which included: legal guardian consent, confirmed PAE, and significant difficulties.

Based on official data, Manitoba had 15,258 births in 2022 [18]. If the population-based prevalence of FASD is 2.5% (mean estimate from 2–3% prevalence), the annual number of new cases of FASD would be 381 (7.3 per week). If the less conservative prevalence rate of 4% is used, the annual number of new cases would be 610 (about 1.7 cases per day, or 11.7 cases per week).

### Northwest Territories

There were two clinics identified in NT capable of conducting neurodevelopmental assessments for FASD. One of these clinics, which opened in 2020, focuses exclusively on adults (over 18 years). One clinic provided data for assessment years 2015–2017. In each year, 10 individuals were assessed for FASD, and 8 to 9 were diagnosed with FASD. In all years combined (2015–2017), a total of 30 individuals were assessed in the one clinic (one person every 12 days) and 26 of them (86.7%) were diagnosed with FASD, one person every 14 days. There were 10 evaluations annually for FASD diagnoses and 45 individuals on the waitlist, in each year. Based on these limited data, the estimated wait time to receive an FASD assessment is approximately 4.5 years. Individuals on this clinic’s waitlist would be referred from health, social or education services. Confirmation of PAE was not required to be on the waitlist, but it was required for the assessment to take place.

The Northwest Territories had 546 births in 2022 [18]. If the population-based prevalence of FASD is 2.5% (mean estimate from 2–3% prevalence), the annual number of new cases of FASD would be 14 per year (1 per month). If the FASD prevalence rate is considered to be 4% (less conservative estimate), the number of new cases would be 22 per year, or 1.8 per month.

### Ontario

A total of 30 clinics were initially identified in ON as capable of conducting neurodevelopmental assessments for FASD. Two of these clinics were closed prior to the start of data collection in October 2018. Of the remaining 28 ON clinics that were contacted, a total of 15 clinics contributed at least one year of data to the survey, resulting in a 53.6% response rate (Table 4).

**Table 4:** Diagnostic capacity of neurodevelopmental clinics (n = 28) in Ontario that participated in the survey (2015–2020).

YEAR OF ASSESSMENT	2015	2016	2017	2018	2019	Totals
**Number of clinics contributing data**	**6**	**7**	**10**	**6**	**8**	
**Individuals assessed in each clinic for FASD diagnoses**						
Mean (SD)	26 (11.2)	45 (22.7)	30 (26.6)	26 (23.4)	42 (32.7)	
Range	7–44	9–78	6–96	8–63	6–106	
** Total (all clinics)**	**158**	**315**	**298**	**157**	**335**	**1,263**
** FASD Assessments/ 10,000 population**	**.12**	**.22**	**.21**	**.11**	**.23**	
**Individuals diagnosed with FASD this year**						
Mean (SD)	18 (9.0)	24 (11.3)	17 (11.9)	16 (16.5)	23 (20.6)	
Range	4–27	5–37	4–40	2–52	0–68	
** Total (all clinics)**	**108**	**169**	**165**	**98**	**182**	**722**
**Diagnostic slots allotted to FASD diagnoses in this timeframe**						
Mean (SD)	24 (18.7)	32 (25.7)	19 (19.3)	31 (32.2)	22 (20.7)	
Range	0–54	0–75	0–60	0–100	0–60	
**Individuals waiting for FASD assessment**						
Mean (SD)	10 (19.8)	49 (52.0)	21 (35.3)	35 (50.3)	45 (41.3)	
Range	0–50	0–120	0–100	0–140	0–96	

On average, 26 to 45 patients were assessed in each diagnostic clinic per year (2 to 4 per month). A total of 1,368 patients were assessed in 2015 to 2019 (114 per month), and over half of them (n = 819; 60%) were diagnosed with FASD (68 per month). This proportion varies across assessment years: diagnoses of FASD occurred in 68.3% of assessments in 2015; 53.6% in 2016; 55.4% in 2017; 62.4% in 2018; 54.3% and in 2019. The average number of diagnostic slots allotted to FASD diagnoses per clinic in this timeframe ranged from 22 to 32 individuals per year. The average number of individuals on the wait list ranged from 10 to 49 per year. Based on information from all clinics 2015–2019, the number of slots is about 26 and number of people waiting for the assessments is approximately 32.

Based on these data, the estimated wait time to get an assessment is approximately one month; however, the descriptions of individual clinics’ waitlists varied in detail provided. Two of the 15 participating clinics noted they did not have formal waiting lists and did not keep track of individuals waiting for assessment. Instead, there was an ongoing process based on client needs and referrals. Four clinics indicated that individuals would be eligible to be on their waitlists following a screening process where either PAE or sentinel facial features are confirmed. Five clinics served children and adolescents exclusively, two of whom specified the age criteria for the waitlist: individuals 21 and under; and individuals 0–6 years old, respectively. Two of these clinics organized assessments by school year, usually assessing only one child per school month (10 months of the year). Only one of these clinics noted waitlist duration, which was between one and two years, depending on a number of factors (e.g., whether a psychological assessment had already been completed). Three clinics in total noted that referrals were organized based on complexity of client needs, age at time of referral and/or assessment resources needed (e.g., if a whole team is required).

Official data indicates that Ontario had 141,699 births in 2022 [18]. If the population-based prevalence of FASD is 2.5% (mean estimate from 2–3% prevalence), the annual number of new cases of FASD would be 3,543 (.7 new cases per day and 68 per week). If the less conservative FASD prevalence rate of 4% is used, the annual number of new cases would be 5,668 (16 per day or 109 per week).

### Summary of capacity and estimate of underdiagnosis rates across all provinces and territories

Table 5 presents diagnostic capacity across provinces and territories. Based on the findings of the current study, a total of 13,404 individuals were assessed for FASD in the five Canadian jurisdictions and 7,408 (55%) were diagnosed with FASD, in 2015–2019. Across all clinics, there were an estimated 14,930 slots assigned to FASD in total across all five years.

**Table 5:** Combined number of diagnoses 2015–2019 and diagnostic capacity per 10,000 in British Columbia, Alberta, Manitoba, Ontario, and Northwest Territories of Canada.

Year of Assessment	2015	Per 10,000	2016	Per 10,000	2017	Per 10,000	2018	Per 10,000	2019	Per 10,000	ALL YEARS TOTAL
**Individuals assessed**	2,576	0.70	2,969	0.81	2,611	0.71	2,389	0.65	2,859	0.78	13,404
**Individuals diagnosed**	1,182	0.32	1,642	0.45	1,592	0.44	1,441	0.39	1,551	0.42	7,408
Proportion diagnosed with FASD (%)	46%		55%		61%		60%		54%		55%
**Slots assigned to FASD**	2,796	0.76	3,173	0.87	2,752	0.75	3,145	0.86	3,064	0.84	14,930

Data source: CAMH REDCap survey and Statistics Canada [18]

Based on information obtained from the clinic survey, we estimated the number of underdiagnosed cases of FASD from birth to 18 years old per year in the participating clinics in selected provinces and territories of Canada (Table 6). Assuming a population-based FASD prevalence rate of 2.5% (conservative estimate) and 4% (less conservative estimate), the percentage of diagnosed cases from birth to 18 years old per year in Alberta is from 2% to 1.2%; British Columbia: 3.5% - 2.2%; Manitoba: 1.8%-1.1%; Ontario: 0.8% - 0.5%; and Northwest Territories: 3.2%-2.0%. Based on the total estimated number of children and youth (from birth to 18 years of age) (115,307) and the number of diagnosed cases per year (1,710) in the participating provinces and territories, the annual FASD diagnostic capacity require at least a 67-fold increase per year, if the select provinces and territories had a goal of diagnosing all children and youth with FASD.

**Table 6:** Estimated number of underdiagnosed cases of FASD from birth to 18 years old per year in Alberta, British Columbia, Manitoba, Ontario, and North West Territories.

		ALBERTA		BRITISH COLUMBIA		MANITOBA		ONTARIO		NORTHWEST TERRITORIES	
**1**	FASD prevalence (%)	**2.5%** ^**a**^	4%** ^**b**^	**2.5%** ^**a**^	**4%** ^**b**^	**2.5%** ^**a**^	**4%** ^**b**^	**2.5%** ^**a**^	**4%** ^**b**^	**2.5%** ^**a**^	**4%** ^**b**^
**2**	Annual birth rate in 2017^c^	53,521	53,521	44,308	44,308	16,803	16,803	140,985	140,985	622	622
**3**	New FASD cases/year^c^	1,338	2,141	1,108	1,772	420	672	3,525	5,639	16	25
**4**	Mean number of diagnosed FASD cases per year^d^	472	472	706	706	137	137	510	510	9	9
**5**	Total number of FASD cases (0 to 18 yrs; assuming the incidence remains the same)^c^	24,084	38,535	19,939	31,902	7,561	12,098	63,443	101,509	280	448
**6**	Number of FASD cases who are diagnosed (0 to 18 yrs)^d^	8,487	8,487	12,708	12,708	2,473	2,473	9,180	9,180	156	156
**7**	Number of FASD cases that are undiagnosed (total number of cases minus diagnosed cases; 0 to 18 yrs)^e^	15,597	30,048	7,231	19,194	5,088	9,625	54,263	92,329	124	292
**8**	Percentage of FASD cases who are diagnosed (0 to 18 yrs) per year	2.0%	1.2%	3.5%	2.2%	1.8%	1.1%	0.8%	0.5%	3.2%	2.0%
**9**	Total cases with FASD (all ages)^f^	107,500	172,000	123,100	196,960	33,924	54,278	351,750	562,800	1,123	1,796

^a^ Popova et al. 2019 [6]

^b^ Flannigan et al. 2018 [13]

^c^ Statistics Canada [14, 18]

^d^ Estimated based on participating clinics in the current study

^e^ Every year the number of undiagnosed cases increases by the number in row 7

^f^ Estimated based on the total population in jurisdiction (Statistics Canada [14])

## Discussion

This study examined the capacity for interdisciplinary team diagnosis of FASD across selected provinces and territories in Canada. Capacity as measured through the number of FASD diagnoses made by reporting clinics has either remained stable or fallen in all jurisdictions studied between 2015 and 2019. The number of diagnostic slots within clinics devoted to FASD assessment rose modestly in some provinces but in no regions has it kept pace with population growth. This study revealed that approximately 98% of cases of FASD are underdiagnosed or misdiagnosed, which is supported by previous publications [15].

The number of FASD diagnoses made across Canada varies a great deal by region. In Ontario, which has a population of approximately 14 million people [14] and an estimated 350,000 to 560,000 people living with FASD, only 722 individuals were reported to receive an FASD diagnosis in this five year period. Based on the data reported from the clinic, as well as the estimated new FASD cases per year in 2022, Ontario needs diagnostic capacity for assessment of 7 new cases every day or 68 per week if FASD prevalence is 2.5%. If the prevalence is 4% (less conservative estimate), the capacity needs to increase to 16 cases per day or 109 per week. Importantly, this is just the clinic capacity needed for diagnosis of the annual birth cohort of new cases. If additional capacity is added for the current undiagnosed cases or just the number of cases on the waiting list, huge increases in capacity are needed. In reality, the majority of individuals with FASD are adults, however, which requires even further expansion of clinic capacity and also capability to diagnose adults and the elderly and to connect them with appropriate support services.

In British Columbia, however, which has less than half that population at about 5 million [14] a total of 1,765 individuals were diagnosed, and in Manitoba with a population of 1.3 million, 687 were diagnosed [14]. Interestingly, both British Columbia and Manitoba have diagnostic services that are provided centrally by one or two large clinics specializing in FASD, whereas Ontario is serviced by many smaller clinics. Together, British Columbia and Manitoba diagnosed 2,452 individuals in 3 clinics while Ontario diagnosed 722 individuals across 15 clinics. If we assume that the number of diagnoses reported here represents only about half of all those made in Ontario because only 53% of clinics in Ontario responded to this survey, the total number of diagnoses would be about 1,347. This yields a rate of .96 diagnoses per 10,000 which is still substantially lower than rates in both British Columbia and Manitoba. This may suggest that regions with large, centralized FASD clinics may be able to deliver improved access and greater capacity overall compared to smaller or multi-functional clinics. Centralized regional clinics in Canada and internationally also offer greater capacity for data linkage, which can help monitor patient outcomes over time and facilitate support services.

Examination of diagnoses made per 10,000 individuals found that although overall capacity has improved somewhat since 2011 [15] there were declines over the period of 2015 to 2019 in at least two provinces, indicating the number of diagnoses being made is not keeping pace with population growth in those areas. Even in provinces such as Ontario where capacity grew over time, the growth appeared unstable and did not seem to reflect a steady progression forward.

If, based on a prevalence rate of 2.5%, there are currently about 913,750 individuals in Canada with FASD this translates to approximately 250 individuals per 10,000 population. Even if every diagnostic slot in the country was filled over the past 5 years and yielded an FASD diagnosis, this would clearly not come close to filling the need for diagnostic services. This study shows that the annual diagnostic capacity in these select provinces and territories require at least a 67-fold increase in FASD diagnostic capacity per year. This demonstrated enormous demand for an increase in capacity requires several major considerations and steps. Firstly, the appropriateness of the current FASD diagnostic guidelines for all age groups (e.g., children under 5 and the elderly) needs to be explored. Facilitating an FASD diagnosis at a younger age is more beneficial on the individual and community levels, but many elderly Canadians remain undiagnosed and may benefit from specialized adult clinics. Secondly, the acceptability and appropriateness of the current multi-disciplinary assessment process can be explored, including the resources required and the acceptability of virtual assessments. Thirdly, the cost-effectiveness of having centralized regional diagnostic clinics (e.g., in British Columbia and Manitoba) versus several smaller clinics, can be explored, and the cost per FASD assessment must be examined for variability across and within regions. Lastly, a feasibility analysis is required to calculate the cost of increasing FASD diagnostic capacity in Canada, and policy implications can be made for which sectors can be leveraged to obtain this funding amidst several competing health priorities.

In addition to limited clinic capacity, underdiagnosis of FASD is likely also related to the strong social stigma associated with the disorder [19], the cost to the health and social systems of funding an interdisciplinary team approach [20], and a lack of training in FASD diagnosis for professionals [21]. The number of referrals made to diagnostic clinics will also be influenced by factors such as primary healthcare providers’ awareness of FASD and the availability of community supports following FASD diagnosis where the presence of these will help to drive demand for diagnostic services.

Guidelines for FASD diagnosis were first published in Canada in 2005 [12] and later updated and revised in 2016 [2]. A stated goal of both versions of the guidelines was to encourage a more consistent interdisciplinary team approach to diagnosis across the country. Despite this, the majority of clinics do not have a complete interdisciplinary team because of limited human and/or financial resources [17]. As well as inconsistent team composition, eligibility criteria and waitlists were found to vary considerably across clinics and provinces. In ON, for example, there is a patchwork of smaller clinics each having unique eligibility criteria and specific age categories served, which leads to gaps and inequities in service. Across provinces and territories, wait times ranged from 1 month to 4.5 years, highlighting inconsistency in timely access to service.

Going forward, there is a need to address gaps in diagnostic capacity across regions, inconsistent wait times, inequities in service accessibility, the need for stable financial investment, and human resource development. Developing regional networks of clinics and centralizing waitlists where possible may assist with equalizing wait times and filling service gaps. Collaboration between regions which have maintained or improved capacity and those which have not may help with disseminating successful strategies.

Limitations of this study include the low response rates in some provinces (ON, AB, NT), which may have limited the accuracy of information, and the lack of investigation into diagnoses made outside of clinic environments. Formal clinics and interdisciplinary groups across provinces and territories were located and contacted for this study, however individual practitioners offering FASD diagnoses were not approached. It is possible that FASD diagnoses were being made by individual physicians alone or with other allied health professionals who conduct their assessments consecutively. Capacity therefore could be higher than estimated here.

The findings of this study will inform policy decisions and advocate for the urgent improvement of FASD diagnostic service capacity and thus, high quality care in Canada. This study can be used as an example for other countries for establishing and/or improving FASD diagnostic services capacity in their regions, and thus, globally.
